# Assertive skills: a comparison of two group interventions with Brazilian university students

**DOI:** 10.1186/s41155-021-00188-7

**Published:** 2021-08-09

**Authors:** Conceição Reis de Sousa, Ricardo da Costa Padovani

**Affiliations:** 1 Curso Psicologia, Instituto Saúde e Sociedade, Universidade Federal de São Paulo (UNIFESP), Santos, Brazil; 2 Departamento de Saúde, Educação e Sociedade, Instituto Saúde e Sociedade, Universidade Federal de São Paulo (UNIFESP), Santos, Brazil

**Keywords:** Assertiveness, Well-being, Rational Emotive Behavior Therapy, Psychoeducation, University

## Abstract

The improvement or acquisition of socioemotional skills contributes to the academic and personal adaptation of university students. The way students think about themselves and others influence their social skills and well-being. Considering the importance of social competence for professional practice in the face of new social realities, the university must invest in programs that promote the socio-emotional development of students. This study compared the effects of interventions based on Rational Emotive Behavior Therapy and Psychoeducation on assertive skills and subjective well-being. This study involved 25 undergraduate students of a public university. The students were randomly allocated to three groups, including the Control group, and they were evaluated by means of questionnaires, inventories, scales, and written evaluation of the group process. The program consisted of 10 meetings and a 6-week follow-up. Irrational beliefs were reduced and their assertive skills’ scores increased in the post-intervention and follow-up evaluations, regardless of the group. Only verbal reports from participants indicated an increase in well-being. The students’ written reports after the end of the meetings indicate that the two forms of intervention were evaluated as promoting change by the students. One of the limitations of the study is the size of the groups. Despite the very small sample size, the study highlights that developing a set of flexible beliefs is fundamental to the exercise of assertiveness.

## Introduction

University admission may be stressful events for some students, especially for those admitted shortly after high school (Bastos et al., 2019; Pedrelli et al., 2015; Reddy et al., 2018). Brazilian university students have few resources to manage their personal and professional relationships, and that can aggravate stress (Ariño & Bardagi, 2018). The National Student Profile Survey of Federal Institutions of Higher Education also indicated that 83.5% of university students have emotional difficulties. It was found that six out of ten students suffer from anxiety and 8.5% of university students are affected by suicidal thinking (FONAPRACE, 2019).

The use of social skills (SS) can partially mitigate the problems of university students in interactions (Lopes, Dascanio, Ferreira, Del Prette & Del Prette, 2017; Del Prette & Del Prette, 2017). Soares et al. (2017) concluded that SS facilitate interpersonal relationships, promoting mental health and professional success of university students. Pereira et al. (2014) found that 43.5% had SS deficits in at least one of the dimensions of the instrument; factor 1 (self-affirmation and coping with risk) had one of the lowest scores, indicating difficulties in defending their rights or respecting those of others among psychology students. Vilela and Lourenço (2019) also pointed out SS deficits in business administration students of a public university. The limitations of the social repertoire students of Exact Sciences can be overcome by social skills training (Lopes, Descanio, Ferreira, Del Prette & Del Prette, 2017).

Assertive behavior can be learned informally or formally through social skill training programs. According to Del Prette and Del Prette (2013), these programs can be supported by different theoretical references such as Behavior Analysis, Bandura Social Learning Theory and Cognitive Approach, such as Rational Emotive Behavior Therapy (REBT).

Programs based on the cognitive approach find that focusing on cognitive elements to be essential to the development of social skills. The absence of a set of rational beliefs (RB) that supports respect for one’s own rights and that of others compromises assertiveness (Caballo, Irurtia & Salazar, 2013). Rational Emotive Behavior Therapy emphasizes changes in the philosophy of life (Rangé, Sousa & Falcone, 2019). According to this theory, overcoming problems requires the abandonment of dogmatic thinking and the adoption of rational/flexible ways of understanding reality.

REBT understands assertiveness deficits as consequences of the activation of irrational beliefs (IB). Assertive people think more flexibly, and their rational beliefs expresses preferences, realistic assessments of adversity, tolerance to frustration and unconditional self-acceptance and acceptance of others and of life itself (Oltean & David, 2018; Rangé et al., 2019).

Subjective well-being (SWB) can be understood as an individual’s self-assessment of satisfaction of their own life, and the prevalence of the experience of positive affects on the negative ones (Layous & Zanon, 2014). Emotional regulation and many other factors can promote SWB (Santana & Gondim, 2016; Wang et al., 2019). Deficits in social skills are commonly correlated with depression, anxiety, learning disabilities, and others (Del Prette & Del Prette, 2017). Experience of these problems can compromise the self-assessment of satisfaction with life and increase the experience of negative affects.

The presence of socially acceptable skills favors academic and personal adaptation in the university context (Van der Zanden et al., 2018). Soares, Porto, et al. (2018) conducted research with university students from private and public institutions that confirmed the relevance of relationship skills for academic success. However, the authors emphasize that even the appropriate academic social behaviors, when accompanied by high demands, can contribute to the emergence of losses in the quality of personal life, such as stress. Therefore, universities must be concerned both with the development of technical competence, as well as with the improvement of interpersonal skills and the promotion of self-knowledge.

Brazilian university students, and those from other countries, have presented psychological distress; actions aimed at promotion and prevention in mental health are necessary (Barkham et al., 2019). There is evidence that skills training programs and psychoeducational interventions have been shown to be effective for this population (Del Prette & Del Prette, 2017). In Brazil, there are few interventions aimed at the development of resources for students to manage the difficulties of higher education (Arenas et al., 2019).

The objective of this study was to compare the effects of two training programs, one based on Rational Emotive Behavior Therapy and the other on Psychoeducation on interpersonal rights, in assertive skills and subjective well-being. The hypothesis was that both programs would promote more flexible ways of thinking and increase assertive skills and well-being. However, the group based on REBT would have better results than the one based on Psychoeducation on interpersonal rights.

## Methods

### Participants

A convenience sample consisted of 25 undergraduate students (24 women and 1 man) from Occupational Therapy (*n*=12, 48%), Psychology (*n*=6, 24%), Nutrition (*n*=5, 20%), and Physical Therapy (*n*=2, 8%). The inclusion criteria were as follows: (a) being enrolled in the first year of the aforementioned courses, (b) not undergoing psychotherapy at the time of intervention and/or using psychotropic drugs, and (c) have availability (day/time) to participate in study.

### Instruments

The following instruments were applied before and after the intervention and the 6-week follow-up: 

1. Students’ Psychosocial Profile Questionnaire (SPPQ). This questionnaire was designed specifically for the present study. The questionnaire included questions about personal and family data, academic life, health, and beliefs about philosophy of life and subjective well-being (e.g., Where did you live before entering university? and “Have you had any significant difficulties or emotional crises in the past 12 months?”)

2. Irrational Beliefs Questionnaire (IBQ). It assesses irrational beliefs as defined by Albert Ellis. This questionnaire was designed by Newmark et al. in 1973 and validated for the Brazilian population by Yoshida and Colugnatti (2002); the studied sample involved young people, university students, of both sexes. The scale is composed of 11 irrational statements such as: “It is horrible when things are not exactly the way we would like them to be,” with which the participant agrees or disagrees. The more positive responses to irrational statements, the more dysfunctional emotions, therefore, the greater the difficulty of adaptation. Cronbach’s alpha coefficient is 0.71.

1. Inventory of Social Skills (ISS-Del-Prette). This instrument was developed by Del-Prette and Del Prette (2001). It assesses the individual’s reactions in situations of social interaction in different environments (e.g., “If a friend abuses my goodwill, I express my displeasure directly”), in which the participant estimates the frequency of their responses in each circumstance using a Likert scale. The instrument produces a general score and five other subscales of social skills: F1 Coping and Self-affirmation with risk, F2 Self-affirmation in the expression of positive feeling, F3 Conversation and Social Development, F4 Self-exposure to strangers and new situations, and F5 Self-control of aggression. Cronbach’s alpha coefficient is 0.75.
2. Life Satisfaction Scale (LSS). Adapted and validated for Brazilian adults. The scale consists of five self-report items on the person’s satisfaction with their living conditions (e.g., “So far I have achieved the important things I want in life”) and is answered on a 7-point Likert scale (Hutz, Zanon, & Bardagi, 2014). Cronbach’s alpha coefficient is 0.91.
3. Positive Affects and Negative Affects Schedule (PANAS). Adapted to the Brazilian context. A self-report scale composed of 10 items that evaluate positive affects (e.g., kind, enthusiastic) and 10 items that evaluate negative affects (e.g., nervous, resentful). The answers are marked on a 1 to 5-point Likert scale (Zanon & Hutz, 2014). Cronbach’s alpha for positive affect coefficient is 0.88 and for negative affect is 0.86.
4. Written evaluation of the group process—it was developed by authors for this study and had as objective to identify the perception of participants in relation to satisfaction with the programs. The instrument was composed by open question about positive and negative points of the group interventions, suggestions for future researches, expectations and objectives achieved (“What were your expectations for the group? Were they covered?”), cognitive and behavioral changes (“Did you notice any change after the meetings? If so, which one (s) and which is the most significant?”), and learning generalization.

### Procedures

Students were recruited on three different occasions: after a lecture on social skills, during a reception event for the new students and via contacts in the classroom. The first author scheduled a meeting with students interested in participating in the study. They were invited to sign an informed consent form and the instruments were applied during a meeting at the university. Participants were randomly divided into three groups. The final distribution was REBT (*n*=12), Psychoeducation (*n*=9), and Control group (*n*=4). This study was approved by the Research Ethics Committee of University.

The meetings occurred simultaneously, coordinated both by the first author of the study and a psychology student. In the first semester, ten meetings were held once a week on consecutive weeks, with an average duration of 60 min in a classroom, during lunchtime.

The interventions in the REBT group focused on the identification and change of irrational/rigid beliefs of each participant, as thinking in a flexible way facilitates the presentation of assertive behaviors. This group focused on improving self-knowledge. In the Psychoeducation group there was a sharing of information about human rights in order to develop a set of beliefs that supported respect for their own interpersonal rights, as well as those of third parties. This group focused on knowledge about the environment (ethics and life values). Both interventions would contribute to the feeling of well-being, as they would improve the perception of satisfaction with life and lead to less frequency in the experience of negative affects.

### Data analysis

In the descriptive analysis, the qualitative variables were summarized in relative frequencies and the data corresponding to the numerical variables were expressed as mean and standard deviation. Comparisons between groups and times were performed using the model of analysis of variance with repeated measures and Bonferroni’s multiple comparisons. The level of significance was set at 5%. All analyses were performed using the R Core Team software.

The participants’ written records about the evaluation of the group process were analyzed qualitatively. The content of the written evaluation went through several readings to define the relevant topics for analysis, based on the objectives of the study.

Six categories were defined a priori: (1) positive points (useful aspects to achieve the group's objectives), (2) negative points (unfavorable conditions to the change process), (3) suggestions for future research, (4) expectations from the program, (5) cognitive changes attributed to participation in the group, and (6) generalization of learning (use of the content learned in the program in other contexts).

## Results

The mean frequency of students’ attendance of the REBT group was 79.16% of the meetings and of the Psychoeducation group, 73.16%. There were no dropouts. The mean age of the students was 19.68 (SD 3.62) years. Only one student was married and only one student was male. Most participants (68%) were originally from other cities in the state of São Paulo, Brazil; 20% already lived in the same city of the *campus*; 4% in another city in the region; and 8% in a city in another state.

Analysis of variance showed time effect for irrational beliefs (p=0.001) and coping and self-affirmation with risk (*p*=0.020). However, there was no interaction effect for the irrational beliefs (*p*=0.291) and for coping and self-affirmation with risk (*p*=0.314).

Table 1 shows the descriptive measures and multiple comparisons between irrational beliefs (IB) and self-affirmation factor and risk coping (F1) in all times (pre- and post-intervention and follow-up).

**Table 1:** Descriptive data and multiple comparisons of the irrational beliefs and self-affirmation factor in all phases (pre- and post-intervention and follow-up)

		REBT	Psychoed.	Control
**IBQ**	T1	3.08 (1.56)	3.22 (1.79)	3.00 (2.00)
	*T2	1.83 (1.40)	1.75 (1.39)	3.00 (1.63)
	*FU	1.83 (1.19)	2.50 (1.77)	2.33 (1.53)
**ISS Factor 1**	T1	8.34 (3.11)	6.49 (2.90)	7.93 (1.22)
	*T2	9.30 (3.13)	8.22 (3.92)	8.61 (2.39)
	*FU	9.90 (3.12)	7.33 (3.32)	7.52 (1.62)

Note. *IBQ* Irrational Beliefs Questionnaire, *ISS* Inventory of Social Skills, *ISS Factor 1* self-affirmation factor and coping with risk, *REBT* Rational Emotional Behavioral Therapy. *p <0.05 compared to T1

Table 1 shows that there was no statistical difference between the groups at different times of the study to irrational beliefs (IB) and self-affirmation factor and risk coping (F1) in all times (pre- and post-intervention and follow-up). However, a difference was observed to both variables among time T1 and times T2 and FU regardless of the group.

Table 2 shows the most frequent irrational beliefs identified in the three groups, at different times of assessment.

**Table 2:** Frequency by irrational beliefs

	REBT			Psychoed.			Control		
Irrational beliefs that made the most sense	T1 (%)	T2 (%)	FU (%)	T1 (%)	T2 (%)	FU (%)	T1 (%)	T2 (%)	FU (%)
1 - It is horrible when things are not exactly the way we would like them to be.	75.0	58.3	53.3	88.9	37.5	62.5	75.0	75.0	66.6
3 - Certain acts are terrible and sinful, and therefore they must be severely punished.	50	25	25	66,7	25,0	37,5	25,0	25,0	33,3

Table 3 shows the results in life satisfaction and negative and positive affects in all phases (pre- and post-intervention and follow-up). There was no statistical difference between the groups at different times of the study. However, a difference was observed to negative affects (Panas negative) among post-intervention (T2) and follow-up (FU) regardless of the group.

**Table 3:** Descriptive and multiple comparisons data of the life satisfaction and positive and negative affects in all phases (pre and post-intervention and follow-up)

		REBT	Psychoed.	Control
**LSS**	T1	23.33 (6.95)	26.63 (5.97)	26.25 (2.22)
	T2	25.67 (5.16)	25.25 (6.58)	28.25 (3.30)
	FU	25.17 (6.16)	27.57 (5.00)	26.67 (2.31)
**PANAS POS.**	T1	27.42 (7.56)	30.78 (4.99)	29.75 (5.85)
	T2	29.92 (4.17)	32.38 (7.46)	31.00 (6.38)
	FU	32.33 (7.74)	29.50 (8.99)	26.67 (7.02)
**PANAS NEG.**	T1	22.08 (8.58)	22.22 (7.68)	21.00 (10.39)
	T2	21.83 (5.10)	22.13 (3.83)	26.00 (7.44)
	*FU	19.75 (8.57)	18.25 (5.26)	17.67 (4.16)

Note. *LSS* Life Satisfaction Scale, *PANAS* Positive and Negative Affect Schedule, *REBT* Rational Emotional Behavioral Therapy. *p <0.05 compared to T2

### Written evaluation of the process

The analysis of the participants’ written evaluation of the group process is presented below:

1. Positive points of the group process that were categorized in two subcategories: (1.1) possibility of meeting other university students and (1.2) having a space to reflect and speak without judgment, as P8 reports: “Openness and confidence about our issues and the whole group, in general, was great. Meetings in addition to causing reflection, stimulated me to make changes in my behavior”.
2. Negative points, of the group process categorized in two subcategories: (2.1) duration of each meeting and (2.2) total number of meetings, for example, P3: “That the meetings are small and reduced in number. Unfortunately, is not an extension [ type of university program]”.
3. Suggestions characterized by ideas to improve interventions, as can be seen in the speech of P7: “Find a way to continue with the experience, create an extension related to this theme”.
4. Expectations in relation to the group, characterized by the evaluation of how much the group process was what it expects at the beginning of the intervention, as P1 reports: “Yes, concede better about assertiveness and how it could help me better deal with my feelings and the relationships I had with people”.
5. Perception of cognitive change due to intervention program, for example, T6: “Yes, I identify the beliefs and reflect on them, I pay attention to the thoughts and control some feelings aimed at assertiveness,” and P6: “Yes, I realized that I started to understand more the abuses I allowed myself to suffer, how to do the tasks of others at school and accept things people don't get hurt/bothered,” and by P8: “These changes started in the first meetings and were gaining strength more and more. A more explicit expression of dissatisfaction and a greater denial of favors that would harm me are the two most significant changes.”
6. Generalization of learning to other environments. P5 mentioned: “I thought of several things when I was on my own. Once I was alone at the bus station, without money and no credit on my phone. I needed to do something. Then, I called a friend collect and asked for help. By attending the meetings, I realized that I could show my ‘weak’ side, in other words, that I needed help, and so I asked. I showed her my feelings, I was crying a lot. I told her what I was feeling and she, and the conversations we had had at the meetings, helped me to think and find a solution.” Only one participant did not mention the use of the resources learned outside the group.

## Discussion

This study compared effects of implementing group training programs based on Rational Emotive Behavior Therapy and Psychoeducation on interpersonal rights, assertiveness, and subjective well-being in university students. The hypothesis was that both programs would promote more flexible ways of thinking and increase assertive skills and well-being, but that the group based on REBT would have better results than the one based on Psychoeducation on interpersonal rights was not confirmed.

However, the high frequency in sessions of both groups (REBT and Psychoeducation) showed the relevance of the interventions. Initial adherence was a challenge, which resulted in the formation of a reduced Control group. The distribution of participants sought to guarantee the number planned for each intervention group at the expense of the number that would constitute the Control group, since dropouts are common, which could compromise the group process.

Another criterion that influenced the distribution between the intervention groups was the availability of the university student in relation to the day of the week on which the group meeting. Some students stated that they preferred not to participate in the study because they were not sure if they should commit to an activity that could take up a lot of their time. This difficulty in having longer commitments, according to Bauman and Raud (2018), may be related to the fear of losing other opportunities. Although extracurricular activities play a central role in the development of professional skills (Ambiel et al., 2019), it is possible that many students did not join the groups because they did not perceive the invitation to participate in the research as an opportunity to acquire or expand important resources for their training. The students who agreed to participate seem to have perceived the meetings as an important part of their training, so much so that in the final evaluation of the meetings, they felt that there was a need for more and longer meetings.

The reduction of irrational beliefs was one of the objectives of this study since they compromise assertiveness and subjective well-being. However, it is not statistically possible to ensure that it resulted only from the interventions. The small number of participants in the groups, especially in the control group, may have interfered with the statistical result.

To understand the way of thinking and acting of university students, it is necessary to know their main beliefs. The three most frequent irrational beliefs involved frustration when things do not happen as desired, avoidance of difficulties, and need to punish a third-party failure. This group of irrational beliefs reveals absolutist ways of thinking and cause emotional distress. This data is in line with meta-analysis study of Oltean and David (2018) that identified an average negative correlation between rational beliefs (related to preferences, non-catastrophizing of events, high tolerance for frustration, and acceptance of oneself, others/life) and psychological distress. Unconditional acceptance of self, others, and/or life play a central role as a protective factor against mental disorders, regardless of age or gender.

Philosophy of life is also composed of beliefs, rational and irrational, that influence expectations regarding the undergraduate course. The participants of this study showed problematic dysfunctional thoughts not only because of their content, but also due to their rigidity regarding the academic performance. This may represent a problem because psychological characteristics such as perfectionism and anger suppression are identified in the literature as risk factors for the mental health of university students (Graner & Cerqueira, 2019).

Some of the participants of this study had rigid beliefs about the need to treat and be treated with justice and consideration and to have comfortable living conditions. These data are consistent with those found by Soares, Leme, et al. (2018), which identified as students’ expectations that the university offered good conditions for professional preparation and for a pleasurable social coexistence.

The belief that it is possible to avoid the discomforts of life is dysfunctional. If a person imagines that they should never feel uncomfortable and that their preferences should materialize most of the time, they will avoid temporary unpleasant situations, even if that decision is detrimental to achieving long-term goals.

After the interventions, the students developed flexible ways of thinking. Those of the REBT group could reduce their self-demand and their “exigencies” from others, as well as to catastrophize less. On the other hand, those who received Psychoeducation became more apt to recognize and question traditional beliefs that used to conflict with the exercise of their legitimate rights. A more flexible thinking can contribute to reduce suffering (Crum, 2019; Oltean & David, 2018). The knowledge of their own beliefs and feelings (REBT group) and interpersonal rights (Psychoeducational group) contributed to the improvement of assertive behavior.

We also sought to expand the social skills of self-affirmation and coping with risk. This result is consistent with that found by Leite-Salgueiro et al. (2018) in a study with university students from northeastern Brazil, which found some good social skills. In our study, it is possible to state that there was an increase in the averages of the variable F1 (self-affirmation and coping with risk) in post-intervention assessments and follow-up in the three groups. So, it is not possible to ensure, statistically, that the changes were produced only by the interventions. The very small number of participants in the control group may have interfered with the statistical result.

On the other side, the written report of the participants shows the perception of benefits of the interventions. Such results corroborate the findings of two studies: (a) a social skill training program with students of Exact Sciences, conducted by Lopes et al. (2017), in which there were gains in SS of self-affirmation and confrontation with risk and self-exposure to strangers, and new situations, and (b) study carried out by Lima et al. (2019), who undertook social skills training with university students from public and private institutions, identifying acquisition of social skills.

The cognitive approach, specifically REBT, allowed the expansion of self-knowledge, the questioning of rigid thinking regarding interpersonal relationships, the recognition of assertive and non-assertive behaviors, and the proposal of practices with the adoption of new interaction patterns (Caballo et al., 2013). On the other hand, in the Psychoeducation-based group, a set of beliefs was constructed that favored respect for their own rights, as well as of third parties. The characteristics of assertive responses were discussed and plans to change non-assertive behaviors were elaborated collaboratively.

In the Control group, there was also a significant change between pre- and post-intervention. Considering that it was a small group, it is possible that if only one of its members oversized its social repertoire, either by a distorted perception of its own behavior or by the desire to make a good impression, this could already affect the result of this group.

The hypothesis that the programs would increase subjective well-being (an individual’s self-assessment of life satisfaction; and the prevalence of the experience of positive affects on the negative ones) has not been statistically confirmed for any of the groups. An individual’s self-assessment of life satisfaction is linked to the achievement of life goals. It is possible that students feel dissatisfied with their lives because they have not yet reached their goals. According to the REBT theory, self-assessment by comparing achievements with those of others or what others think (self-esteem) leads to dissatisfaction. On the other hand, self-acceptance and acceptance of failures promote health (Dryden & Ellis, 2006). In the meetings, the students had difficulty accepting their possible mistakes regarding academic performance and self-image, indicating a rigid thinking.

In the REBT group, the development of a healthy life philosophy was instigated, guided by preferences and not by “tyrannies.” This concept of REBT is compatible with the concept of self-compassion, which refers to the adoption of an attitude of respect with oneself, instead of a severely critical posture, in the face of its flaws, as well as the acceptance of negative thoughts and emotions (Neff & Vonk, 2009). A survey of 184 university students conducted by Stephenson et al. (2018) found that irrational beliefs correlate negatively with self-compassion and positively with self-esteem. This study also pointed out that self-compassion favors mental health, while attempts to maintain self-esteem are linked to the experience of depression and anxiety.

In the Psychoeducation group, the responsibility for changing, or maintaining, beliefs that were incompatible with self-acceptance and the exercise of assertive skills was instigated.

The two programs in our study proposed interventions that made it possible to question the concept of self-esteem and strengthen students’ self-acceptance to increase their perception of satisfaction with life.

Another point that may have compromised the well-being of students was the distance from family and friends, as 76% of the participants lived outside the city of origin. Based on a study on sociability, Sposito, Nakano and Chen (2016) point out perceptions and values when comparing young Brazilian and Chinese university students. According to the authors, students from both countries use most of their free time at home to access the Internet. Another interesting fact about this comparative study, involving both countries, is that Brazilian students mentioned a conversation with parents and other family members as a frequent activity, which shows the importance attributed to living with the family in the same house.

The written records of participants (both groups) show that the participants felt more satisfied with their life after the interventions. The students recognized the development of resources that favored their interpersonal relationships, as well as self-knowledge, which may have increased the subjective well-being.

It was not possible to confirm the hypothesis that the REBT group would be more effective than the Psychoeducation group for promoting assertive skills and subjective well-being. The duration and number of meetings may have been insufficient for evidencing the effects of cognitive changes.

Murta and Trócoli (2009) point out some factors, which may have also influenced our results, to explain the similarity of the results when analyzing the similarity of the effects of two different interventions for occupational stress management, namely (a) the small groups, since the smaller the sample, the lower the sensitivity of a statistical test; (b) dissemination of information between the groups, since the students met in their classes and residences; and (c) the similar way of fostering the construction of a new belief system, because the researcher conducts both groups.

Finally, it is necessary to reflect on the impact of interventions based on qualitative data on satisfaction with the group process and the resources developed in the group. Students from both groups highlighted as positive points: (a) the possibility of meeting other university students, which may be related to a group process aspect: universality, that is the discovery that different people may suffer from the same types of problems, and (b) having a space to reflect and speak without judgment: the climate of acceptance, trust, and support and the sharing of knowledge (based on REBT or Psychoeducation) seem to have been indicated by students as an important factor to facilitate changes. On the other hand, the duration of each meeting and the number of meetings were assessed as insufficient to consolidate the changes. The students’ suggestions were to expand the exchange space, which may indicate their interest in developing or improving their personal resources. The main expectation of the students was to expand their knowledge about themselves and about interpersonal relationships in order to develop new ways of relating. This may indicate that those entering university think about the importance of developing technical and personal knowledge.

Regarding the category related to the perception that the meetings produced changes in the way of thinking and acting, it is necessary to highlight beliefs considered by the participants as difficult to change: the idea that it is unacceptable to fail or that others fail. Throughout the meetings, students were able to understand the role of beliefs in their behavior and emotions. This knowledge helped them to overcome the deficits of assertiveness and to see life in a more positive way. Recognizing that everyone can fail (assertive ability) seems to have contributed to making the way they perceive their interpersonal problems more accurate, which can contribute to their resolution.

In the category related to the impact of the intervention on daily relationships, characterized by the use of learning outside the group, the students pointed out that the new resources were important in their personal and academic-professional context. Whereas only competence technique is insufficient to deal with the demands of the job market, the university needs to offer programs that favor the development of interpersonal skills.

The interventions facilitated the strengthening of ties between participants and outside groups. Such was the appreciation of the collective space to share problems and seek solutions that the students of the Psychoeducation-based group continued to meet fortnightly, even in the absence of the researcher.

## Limitations

One of the limitations of this study is the very small number of participants and the fact that only one man participates in the three groups, thus compromising the possibility of generalizing the results. The difficulty in getting university students who agreed to make a commitment to research jeopardized the formation of the Control group. Another limitation of the study is that external validation by judges and peers was not used to analyze the content categories of the written assessments of the group process.

The present study was developed only with university students from a public institution in southeast Brazil. It is important that new investigations include students from private institutions in other regions of the country.

## Conclusions

The hypothesis that the group Rational Emotive Behavior Therapy, when compared to that of Psychoeducation, would promote greater changes in assertive behavior and subjective well-being among Brazilian university students has not been confirmed. It was verified statistical differences in irrational beliefs and coping and self-affirmation with risk, regardless of the group. It is possible that the small sample size did not lead to a statistically significant difference.

Future research may extend the duration and number of meetings in order to favor the consolidation of flexible beliefs and exercise of assertiveness, perform the sample calculation, and use external validation by judges and peers to explore qualitative data.

Another point to be reconsidered is the moment that the interpersonal skills program is offered. Upon entering the institution, students find many stimuli and are still unaware of the importance of developing their interpersonal resources. This condition may decrease their interest in joining this type of program.

Regarding irrational beliefs, it was observed that three most frequent irrational beliefs had as main content frustration, avoidance, and punishment. Learn to be flexible and assertive are fundamental skills for building healthy relationships and for well-being. Universities play an important social role in formation of citizenship. In this perspective, the university must to incentive the development of projects that promotes the psychosocial development of university students.

One important point to consider in this study was the active posture and interest of participants to learn assertive behavior and develop personal resources. Considering the need for interventions aimed at promoting mental health in a university environment, accentuated by the effects of the COVID-19 pandemic, this study highlights the role of recognizing one’s irrational beliefs (REBT group) and questioning about the belief system that supports assertiveness (Psychoeducation group) as resources to live better with yourself and with others.

## Data Availability

The datasets used and/or analyzed during the current study are available from the corresponding author on reasonable request.

## Abbreviations

F1: Self-affirmation factor and coping with risk
ISS-Del-Prette: Inventory of Social Skills
LSS: Life Satisfaction Scale
IR: Irrational beliefs
IBQ: Irrational Beliefs Questionnaire
PANAS: Positive Affects and Negative Affects Schedule
RB: Rational beliefs
REBT: Rational Emotive Behavior Therapy
SS: Social skills
SPPQ: Students’ Psychosocial Profile Questionnaire
SWB: Subjective well-being
